# Feature engineering for MEDLINE citation categorization with MeSH

**DOI:** 10.1186/s12859-015-0539-7

**Published:** 2015-04-08

**Authors:** Antonio Jose Jimeno Yepes, Laura Plaza, Jorge Carrillo-de-Albornoz, James G Mork, Alan R Aronson

**Affiliations:** 1 Department of Computing and Information Systems, The University of Melbourne, Parkville, 3010 VIC Australia; 2 National Library of Medicine, 8600 Rockville Pike, Bethesda, MD 20894 USA; 3 UNED NLP & IR Group, C/Juan del Rosal 16, Madrid, 28040 Spain

**Keywords:** Text categorization, Feature engineering, Biomedical literature, MeSH indexing

## Abstract

**Background:**

Research in biomedical text categorization has mostly used the *bag-of-words* representation. Other more sophisticated representations of text based on syntactic, semantic and argumentative properties have been less studied. In this paper, we evaluate the impact of different text representations of biomedical texts as features for reproducing the MeSH annotations of some of the most frequent MeSH headings. In addition to unigrams and bigrams, these features include noun phrases, citation meta-data, citation structure, and semantic annotation of the citations.

**Results:**

Traditional features like unigrams and bigrams exhibit strong performance compared to other feature sets. Little or no improvement is obtained when using meta-data or citation structure. Noun phrases are too sparse and thus have lower performance compared to more traditional features. Conceptual annotation of the texts by MetaMap shows similar performance compared to unigrams, but adding concepts from the UMLS taxonomy does not improve the performance of using only mapped concepts. The combination of all the features performs largely better than any individual feature set considered. In addition, this combination improves the performance of a state-of-the-art MeSH indexer. Concerning the machine learning algorithms, we find that those that are more resilient to class imbalance largely obtain better performance.

**Conclusions:**

We conclude that even though traditional features such as unigrams and bigrams have strong performance compared to other features, it is possible to combine them to effectively improve the performance of the *bag-of-words* representation. We have also found that the combination of the learning algorithm and feature sets has an influence in the overall performance of the system. Moreover, using learning algorithms resilient to class imbalance largely improves performance. However, when using a large set of features, consideration needs to be taken with algorithms due to the risk of over-fitting. Specific combinations of learning algorithms and features for individual MeSH headings could further increase the performance of an indexing system.

**Electronic supplementary material:**

The online version of this article (doi:10.1186/s12859-015-0539-7) contains supplementary material, which is available to authorized users.

## Background

Text categorization is the task of automatically assigning pre-defined labels to text [1]. Even though several methods can be used, machine learning is appealing due to the large data sets that are available as training data that allow for automating the development of categorization models effectively.

In the biomedical domain, research in automatic text classification is usually conducted in the context of indexing MEDLINE^®;^ citations with MeSH^®;^ descriptors. There are over 23 M citations in MEDLINE, with a growth rate of over 800 k new citations every year. This growth rate makes it difficult to keep up-to-date with new discoveries. To help cataloguing and searching biomedical documents, the US National Library of Medicine (NLM)^®;^ has produced the medical subject headings (MeSH) controlled vocabulary. Each MEDLINE citation is manually assigned a number of relevant medical subject headings that classify the document according to its topic. Manual indexing is, however, costly. As stated in [2], MEDLINE indexing is the responsibility of a relatively small group of highly qualified indexing contractors and staff at the NLM who find it difficult to maintain the quality of this huge resource. In this situation, automatic methods to categorize citations might be relevant.

Current work on automatic biomedical text categorization based on machine learning has been dealing largely with *bag-of-words* representations. *Bag-of-words* based on unigrams are easy and fast to build and have shown good performance on many text categorization tasks [3]. However, specialized domains such as the biomedical one suffer from a varied terminology which might be too sparse to efficiently train machine learning models. For instance, when building a categorization rule for *prostate cancer* documents, there are terms with low frequency that are highly relevant for classification, e.g. *prostate adenocarcinoma*. Ontologies and terminological resources like the Unified Medical Language System (UMLS^®;^) [4] can help in the normalization of terms found in text and could be used to derive additional feature sets (e.g., through synonymy and generalization). This normalization has already been proposed for indexing MEDLINE with MeSH controlled vocabulary in the *Restrict-to-MESH algorithm* [5].

There is also information latent in the structure of the text that is not usually considered in the categorization task, i.e., if the terms come from a specific section of a citation. For instance, if *prostate cancer* is mentioned only in the “Background” section, then it is not as relevant as if it is found in the title of the article. This information could be used by the classifier in order to build a better categorization model, as has already been shown useful for the retrieval [6] and the selection of topic relevant sentences [7-9].

In addition, there is meta-data that is usually linked to biomedical citations that is not taken into account and could be used to provide a better categorization performance. For instance, there are specialized journals in which certain types of articles are published, e.g. specialized journals for breast cancer like *Breast Cancer Research*.

Other issues with the features used in text classification are not considered in this work. For instance, there are learning algorithms that benefit from feature selection or better tuning of weights used to represent the features [10]. In previous work, several machine learning (ML) approaches and their combination [11] have been evaluated within the biomedical domain, trying to reproduce, for instance, MeSH indexing. On the other hand, most previous work relies on comparing or combining several ML algorithms (Bayesian models, neural networks, decision trees, regression, etc.) on *bag-of-words* representations. Little attention has been given to measuring the impact of feature engineering.

The motivation of this work is to evaluate the performance of machine learning algorithms on several types of features in the context of text categorization (in particular, categorization of MEDLINE citations with different MeSH headings), testing representations based on lexical, syntactic, semantic and argumentative information. In addition to evaluating each individual feature set, we test different combinations of features. We also evaluate several machine learning approaches to overcome the imbalance between the small number of positives versus the large number of negatives.

Overall, we find that the *bag-of-words* representation is a competitive baseline, slightly improved by the use of higher order n-grams. We also find that the combination of different features outperforms the *bag-of-word* baseline. In addition, a large improvement is obtained by using techniques that target the imbalance in the training set. Finally, we observe that the performance may vary depending on the evaluated MeSH heading, which might imply that optimal performance would require selecting a specific feature set in some particular cases.

## Related work

The most frequently used feature model is the so called *bag-of-words*: each position in the input feature vector corresponds to a given word or phrase and stores either a binary value indicating its presence or a numerical value indicating its frequency or even its TF-IDF [12,13]. Other authors have used phrases rather than individual words [14,15], but the results obtained are not significantly better (or are even worse) than those of the *bag-of-words* approach. Representations based on named entities have also been explored. They have proved to be useful when a reduction of the number of features is needed [3], but do not add any classification power.

There is previous work on feature engineering for text categorization not bound to the biomedical domain [3] that examines a large set of text representations, including lexical (e.g., *bag-of-words*), syntactic (e.g., noun phrases and key phrases) and semantic features (e.g., synonym and hypernym relations and concepts from WordNet). Syntactic features have also been used in combination with other lexical or semantic ones.

Previous work has attempted to improve classification by using bigrams as features [16]. While using bigrams together with unigrams has proved to be potentially beneficial for text classification, using bigrams alone leads in most cases to a decrease in results comparing with the use of *bags-of-words*.

Lewis [17] was the first to study the effect of syntactic phrases in text categorization, and found that a Naïve Bayesian classifier with only noun phrases yields significantly lower results than a standard classifier based on *bag-of-words*.

In [18], part-of-speech tags are appended to words in the feature vector, and the position of each word in the text (whether it appeared in the first quarter, last quarter, or middle half of the document) is also codified. None of these features, however, improves significantly from using unigrams alone. Furnkranz et al. [19] used automatically extracted linguistic patterns as features to a Naïve Bayesian classifier, and got a consistent improvement in precision. More recent efforts have proposed the use of external resources in order to semantically enrich the feature sets. In [20], for instance, common-sense and domain-specific knowledge are used to enrich the *bag-of-words*, using repositories of knowledge such as the Open Directory Project (ODP), Yahoo! Web Directory, and the Wikipedia encyclopedia.

### Text categorization in the biomedical domain

In the biomedical domain, text categorization is usually studied within the task of indexing MEDLINE citations with headings from the MeSH controlled vocabulary. This indexing provides an efficient way of accessing and organizing the huge volume of biomedical publications in MEDLINE. MEDLINE indexing is performed manually at the NLM and is supported by automatic tools provided by the NLM Indexing Initiative [21]. MeSH contains more than 26 k terms in 2013; thus the indexing of MEDLINE with MeSH provides a large set of data to train and evaluate automatic categorization methods.

A good number of features have been already considered for MeSH text classification. In [22], MEDLINE citations are represented using all single words and bigrams found in the titles and abstracts of the citations, after stop word removal. Documents are represented by vectors of binary attributes indicating the presence or absence of the features. Feature selection is used to reduce the feature dimensionality. Yetisgen and Pratt [23] evaluated phrases in addition to the *bag-of-words* representation on the OHSUMED set [24], a clinically-oriented MEDLINE subset over a five-year period (1987-1991) with 348,566 references. They found that combining both representations improved the performance of any single representation.

In [25,26], text categorization is based on the references of related work made by the text itself to assign MeSH descriptors to a new citation. These approaches make use of the keywords manually assigned to the documents that are cited in the target document. A problem of this approach is that, when applied to the classification of MEDLINE citations, references are not usually available.

In [27], a graph-based ranking algorithm, called MEDRank, is presented that models the text as a graph of UMLS concepts, and identifies the most “central” concepts within the graph. These central concepts are considered to be good indexing terms and finally translated to the MeSH vocabulary.

In the BioASQ challenge [28], the participants had to deliver MeSH indexing suggestions for a set of new, thus not previously indexed, citations. Participants of the challenge found that bigrams seemed to be more effective than unigrams. Several methods used feature selection without achieving any improvement over not using it; other methods used the taxonomical structure from MeSH, that purely encodes an *is-a* relation between the headings. Many interesting submissions were done with ideas about which features could be used and experiments that showed the negative effect of applying feature selection [29]. The submissions from the participants were measured against the performance of the Medical Text Indexer (MTI) system [21,30], that was considered as a baseline of the performance of the participating systems.

More advanced approaches have considered a change in the representation of the documents, by training first-order logic models based on Inductive Logic Programming (ILP). In a more general domain, we find the work of Cohen [31]. It has also been considered for biomedical indexing [32].

### The medical text indexer

The NLM Indexing Initiative has developed the MTI, which is a support tool for assisting indexers as they add MeSH indexing to MEDLINE. Given a MEDLINE citation with only the title and abstract, MTI will deliver a ranked list of MeSH headings. MTI has two main components: MetaMap [33-35] and the PubMed Related Citations (PRC) algorithm [36]. MetaMap indexing (MMI) annotates citations with UMLS concepts. UMLS concepts are next mapped to MeSH following the Restrict-to-MeSH [37] approach which is based primarily on the semantic relationships among UMLS concepts. The PRC algorithm is a modified k-Nearest Neighbours (k-NN) algorithm that proposes indexing candidates for MeSH headings which are not always explicitly present in the title and abstract of the citation but which are used in similar contexts. The citation being indexed by MTI is not considered when running it through MTI based on its PubMed identifier (PMID).

In a process called Clustering and Ranking, the output of MMI and PRC are merged by linear combination of their indexing confidence. The ranked lists of MeSH headings produced by all of the methods described so far must be clustered into a single, final list of recommended indexing terms. The task here is to provide a weighting of the confidence in the assignment.

Once all of the recommendations are ranked and selected, a post-processing step validates the recommendations based on the targeted end-user. The main goal of this step is to ensure that the proposed indexing adheres to the NLM’s indexing polices. This step applies a set of specific rules triggered by either headings or terms from the text.

## Feature types

In this section, we present the different features used to represent the MEDLINE citations for MeSH indexing based on machine learning.

### Bag-of-words (Unigrams)

As already mentioned, this is the most widely used feature type for text classification. In the *bag-of-words* representation, each word corresponds to a feature with a weight assigned to it. This weight is usually the number of times that the word occurs in the document or a binary value indicating its presence. In our experiments, we test both representations: **frequency** and **presence (binary)**. We have used a standard stop word list and a frequency threshold to filter out tokens.

### Linguistic features

As stated in [3], *bag-of-words* representations discard a great amount of information from the original documents, since word order and syntactic structures are broken. To deal with this drawback, more complex representations based on n-grams and phrases may be used. In particular, we use the following features:

- **Bigrams:** Previous work in categorization of general-domain texts has shown that the use of bigrams can substantially raise the quality of feature sets [38]. We have generated a representation that combines bigrams (two continuous tokens) and unigrams.
- **Noun phrases:** Even though different experiments [12,17] have found that the use of phrases as classification features causes a decrease in performance, we still believe it is worth testing them in the context of biomedical text categorization. To identify noun phrases, we use MetaMap [33-35]. MetaMap is a tool created by the NLM that maps text to UMLS Metathesaurus^®;^ concepts [39,40]. MetaMap uses a variation on the MedPost Tagger [41] to assign syntactic parts of speech and then uses the tags to identify phrases.

### Citation meta-data

Citations in MEDLINE contain meta-data about the citation that is already there before indexing. It has been shown that this additional meta-data can improve classification performance in the case of indexing Publication Types (PT) [42]. We test the following meta-data from the MEDLINE citations as classification features:

- **Journal**: The journal in which the publication has been published may give some insights on the main topic of the citation. An internal study within the NLM [43] found that, over the last five years and for 6,600 journals under study, the average usage of unique MeSH terms to index citations was only 999 out of 27,149. The study also revealed that each journal seems to have a small subset of MeSH terms that it focuses on. The maximum number of MeSH Headings (MHs) used by a single journal was 17,501 of the 27,149 (based on 58,032 articles). The fewest MHs used by a single journal was 3 of the 27,149 (based on 2 articles). Providing an overall average of 999. We have used the *NlmUniqueID* tag that provides a unique journal identifier instead of using the journal name that is not consistently defined.
- **Authors**: Since researchers are usually specialized in specific topics, they can help to predict the MeSH indexing terms relevant to the citation.
- **Affiliation of authors**: Since research groups usually work in a limited set of areas, they can also help to improve indexing performance.

### Concept-based representation

Concept-based representations have been previously used in general purpose text categorization [3,44]. Concept-based representations are expected to better model the meaning of the text, by capturing semantic relations between words (such as synonymy) and avoiding word ambiguity. We use UMLS Metathesaurus concepts as classification features. Concepts are retrieved using the MetaMap tool. MetaMap is invoked using the -y flag that uses the default word sense disambiguation algorithm provided in Metamap. We test two different representations based on UMLS Metathesaurus concepts:

- **Frequency of CUIs**. We use the concept unique identifiers returned by MetaMap as features.
- **Frequency of concepts’ names**. Instead of using the CUIs, we use the concepts’ names. Note that concept names are not necessarily unique, so that different concepts with the same name will be represented by the same feature, so that ambiguity issues may arise.

### Hypernym-based representation

As in [3], we also test representing documents at a higher-level of generalization. This may allow for (a) a better representation of the semantics of the documents, by capturing the semantic relationships between words, and (b) the neutralization of the effect of infrequent but important terms.

For each of the UMLS Metathesaurus concepts that are returned by MetaMap, we retrieve from the UMLS its hierarchy of hypernyms. We next build different feature vectors by taking hypernyms at different levels:

- **First level taxonomy**: The feature vector is composed of the parents of the UMLS concepts that are found in the citation.
- **Second level taxonomy**: The feature vector is composed of the grandparents of the UMLS concepts that are found in the citation.
- **Third level taxonomy**: The feature vector is composed of the great-grandparents of the UMLS concepts that are found in the citation.

To represent these features, we have considered only the presence of each feature in the document.

### Argumentative structure

Using information about the argumentative structure of MEDLINE abstracts has been shown to be of use in different tasks [8,9], such as information retrieval [7,45,46] and automatic summarization [47]. The hypothesis is that different sections of an abstract will include different types of information, so that information in some sections may be more relevant for MeSH indexing than that in other sections.

MEDLINE contains structured abstracts that can provide argumentative labels. A structured abstract is an abstract with distinct labeled sections (e.g., Introduction, Background, or Results). From 2010, the labeled sections in MEDLINE structured abstracts are mapped to the US NLM categories: OBJECTIVE, CONCLUSIONS, RESULTS, METHODS, and BACKGROUND [48].

We use these argumentative labels in the abstracts as classification features in combination with the *bag-of-words* representation, by adding to the different words information about the sections of the abstract in which they appear. Since not all abstracts in MEDLINE are structured, argumentative labels for non-structured abstracts are obtained automatically, by using a logistic regression model trained using structured abstracts as developed in [8,9].

### Title versus abstract information

Closely related to the argumentative features described above, here we distinguish between features from the title of the citation and features from the abstract. The title given to a document by its author is intended to represent the most significant information in the document, and thus the words within it are expected to be the most meaningful words that describe the content of the document [49].

We test whether making a distinction between features extracted from the title and features extracted from the abstract may improve classification performance.

### MTI derived features

As introduced above, MTI is used to support indexing MEDLINE citations and has been found to produce very competitive results. The MeSH headings suggested by MTI are used as features for the learning algorithm.

In the evaluation, we consider the current MTI configuration as a baseline system; for PRC this means selecting MeSH headings appearing at least 4 times or more in the top 10 citations recovered from MEDLINE using the Related Citations algorithm [36]. MTI combines the MMI and PRC components that includes additional *ad-hoc* rules added to either comply with indexing policies or address indexers’ feedback.

In the experiments, we have used MTI output as features for the learning algorithm consisting of the MeSH headings predicted by MMI and PRC algorithms.

## Experimental setup

### Data sets

The evaluation data set is based on the data set previously used in [11] and available from the NLM II website [50]. The data set a subset of MEDLINE citations that completed the indexing process between November 2012 and February 2013. We have considered citations within a short period of time to ensure that there were no problems with policy changes in indexing that would have posed problems with the learning algorithms. As a result, our evaluation collection consists of 143,853 citations, 94,942 are used for training and 48,911 are used for testing.

From this set, we selected MeSH headings with at least 1,500 citations indexed. The number of selected MeSH headings is 63. We randomly selected 50 MeSH headings (see Table 1) out of these 63 MeSH headings. The training set contains 21,927 of the possible 27,149 MeSH headings in the 2014 MeSH vocabulary.

**Table 1:** **MeSH descriptors in the evaluation collection, and their citations frequencies in the training set**

**Descriptor**	**Frequency**	**Descriptor**	**Frequency**
Humans	66612	Disease models, Animal	2203
Male	39007	Rats, Sprague-Dawley	2160
Female	38793	Sensitivity and specificity	2155
Animals	25529	Cell proliferation	2124
Adult	21471	Biological markers	2088
Middle aged	20867	Cohort studies	2072
Young adult	9512	Risk assessment	2049
Adolescent	8869	Brain	2035
Mice	7980	Mutation	2025
Treatment outcome	6749	Mice, Inbred C57BL	2005
Aged, 80 and over	6015	Cell line	1947
Child	5759	Apoptosis	1901
Rats	5610	Infant, Newborn	1865
Risk factors	4896	Tomography, X-Ray computed	1862
Prospective studies	3178	RNA, Messenger	1843
Questionnaires	3064	Age factors	1763
Signal transduction	2925	Algorithms	1698
Cell line, Tumor	2911	Models, Molecular	1692
Molecular sequence data	2695	Antineoplastic agents	1681
Pregnancy	2672	Gene expression regulation	1669
Infant	2551	Dose-response relationship, Drug	1627
Magnetic resonance imaging	2545	Amino acid sequence	1625
Cells, Cultured	2451	Genotype	1561
Prognosis	2450	Neoplasms	1521
Case-Control studies	2383	Phylogeny	1518

### Machine learning algorithms

The assignment of MeSH descriptors to citations is a multi-label classification problem, since more than one descriptor may be assigned to a document. We have dealt with each category or label as a binary classification problem. For each MeSH heading a classifier is built that decides whether the document should be assigned to the corresponding class.

We have used different learning algorithms with the various feature sets described in previous sections. We have used two learning algorithms that have shown competitive results in previous work. The first one is Support Vector Machine. We have used the implementation available from SVM light [51]. Default options for SVM light were used, i.e. linear kernel and a C parameter set to $\frac {1}{\text {average} X \cdot X}$, thus estimated based on the feature representation being used. The number of features per representation is available from Additional file 1: Extended results. The second one is AdaBoostM1 that uses a C4.5 decision tree as the base learner with pruning confidence set to 0.25. We have used the implementation available from the MTI_ML package [52], that has reported good results for MeSH indexing.

As we observe in Table 1 there is a large imbalance between the citations indexed with a given MeSH heading (positives) and the citations not indexed with it (negatives). This is a problem for learning algorithms that optimise learning for accuracy, and in some cases the trained models simply predict the majority class. We have evaluated using AdaBoostM1 with oversampling to provide more weight to the positive citations. We have evaluated as well using SVM-perf optimizing F-measure instead of accuracy [53], using the -c parameter to trade-off between training error and margin. Finally, we have used the implementation available from [54] and the -c value has been set to 100.

### Evaluation metrics

To evaluate the different feature sets, we use *precision*, *recall* and *F-measure*, as traditionally done in supervised classification. The F-measure is the harmonic mean of precision and recall, and is computed as follows:

(1) $$ F-measure = \frac{2 \times recall \times precision}{recall + precision}  $$

(2) $$ precision = \frac{true \; positive}{true \; positive + false \; positive}  $$

(3) $$ recall = \frac{true \; positive}{true \; positive + false \; negative}  $$

where true positive is the number of the citations correctly assigned to the MeSH category, false positive is the number of citations incorrectly assigned to the MeSH category, false negative is the number of citations incorrectly rejected from the MeSH category, and true negative is the number of citations correctly rejected from the MeSH category.

Average results have been provided for the 50 headings in the set *C*. Micro average sums all the true positives (TP), false negatives (FN) and false positives (FP) as shown in formula 4. These values are then used to calculate precision, recall and F-measure.

(4) $$ \begin{aligned} {}{TP}_{micro}\,=\,\sum\limits_{i \in C} {TP}_{i} \quad {FP}_{micro} = \sum\limits_{i \in C} {FP}_{i} \quad {FN}_{micro}=\sum\limits_{i \in C} {FN}_{i}  \end{aligned}  $$

(5) $$ {\fontsize{8.5pt}{9.6pt}\selectfont{\begin{aligned} {}{precision}_{micro} \,=\, \frac{{TP}_{micro}}{{TP}_{micro} + {FP}_{micro}} \,\,\,\, {recall}_{micro} \,=\, \frac{{TP}_{micro}}{{TP}_{micro} + {FN}_{micro}} \end{aligned}}}  $$

Macro averages are calculated averaging the precision and recall calculated for each individual category. F-measure is then calculated based on this average.

## Results

Table 2 shows the average classification performance for several learning algorithms and several feature sets, in terms of F-measure. Unless otherwise specified, macro average values are used since very frequent categories will have more relevance in micro averaging. Extended results are available from Additional file 1: Extended results (including results per MeSH heading, recall and precision and micro and macro averages). Statistical significance of the results was computed using a randomization version of the two sample t-test [55].

**Table 2:** **Feature comparison over all results**

**Feature**	**SVMLight**	**SVM-perf**	**AdaBoostM1**	**Ada Over**
Unigram	0.418	0.492 *†*	0.420	0.471 *†*
Bigram	0.406	0.513* *†*	0.420	0.477* *†*
Argumentative	0.403	0.479 *†*	0.415	0.464 *†*
Noun phrases	0.222	0.329 *†*	0.222	0.271 *†*
Concepts	0.409	0.497* *†*	0.427	0.480* *†*
CUIs	0.398	0.496	0.422	0.475 *†*
MTI predictions	0.513*	0.531* *†*	0.478*	0.501* *†*
MTI MMI	0.398	0.454 *†*	0.367	0.382 *†*
MTI PRC	0.481*	0.502 *†*	0.430	0.453 *†*
First level taxonomy	0.300	0.456 *†*	0.351	0.429 *†*
Second level taxonomy	0.222	0.424 *†*	0.329	0.393 *†*
Third level taxonomy	0.173	0.383	0.285	0.341 *†*
Journal	0.115	0.193 *†*	0.126	0.208 *†*
Affiliation	0.046	0.064	0.045	0.044 *†*
Author	0.062	0.137 *†*	0.081	0.084 *†*

Results are reported in F-measure. Binary representation of features is used. Several learning algorithms have been used including SVMLight, SVM-perf, AdaBoostM1 and AdaBoostM1 with oversampling of positive instances (Ada Over). For each column, results significantly better than unigram (p >0.05) are indicated with *. For each pair of methods (SVMLight/SVM-perf and AdaBoostM1/Ada Over), statistical differences are highlighted using *†*.

### Machine learning algorithms

Concerning the ML algorithms, our experiments provided some interesting conclusions (see Table 2). Overall we can see that AdaBoostM1 with oversampling and SVM optimized for multi-variate measures perform much better than AdaBoostM1 and SVM, which is significant. Using SVM optimized for F-measure improves the performance over other learning algorithms on the same set of features, usually improving recall at the cost of precision for the configuration of the algorithm, which is not significantly better compared to AdaBoostM1 with oversampling. AdaBoostM1 improves when using oversampling on the data set before the training.

### Feature sets

Concerning the feature sets, it can be observed from Table 2 that the best performance is obtained when simple and traditional features such as **unigrams and bigrams** are used; the difference is significant except for AdaBoostM1. Note that, in this table, binary representation is used. More specifically, bigrams are the best performing individual features. Just as a reminder, in our implementation bigrams include unigrams as well. Bigrams boost the precision at the cost of some recall, even though the average performance does not change. A close look at the results by MeSH heading (see Additional file 1: Extended results) shows that bigrams perform better on the most frequent MeSH headings, but performance drops in the less frequent ones.

Our results corroborate previous claims that the **use of noun phrases** as classification features causes a significant decrease in performance compared with the use of unigrams [17]. This is in fact one of the worst performing classification features we have tested. Lewis [17] suggested that the main reasons for these results are that phrase-based representations (i) have an uneven distribution of feature values and (ii) contain many redundant features.

**Concept representation** of the citations has performance comparable to the unigrams, being even better for some of the learning algorithms, even though the differences are in most cases not significant. This is the second best performance feature, after bigrams. Surprisingly, we obtain better results when representing concepts by their name than when representing them by their CUI. This may be due to the fact that MetaMap may be incurring errors when solving ambiguity to assign the concept unique identifiers.

The results from the **hypernym representations** are, however, very disappointing. Generalization of concepts at the different levels has showed a significant decrease in performance compared with using the concepts themselves. We observe that the higher the generalization, the worse the classification results.

On the other hand, using the **meta-data** from the MEDLINE citations (journal, authors and affiliation) as the only features is not enough to correctly classify the citations. This was expected, since they provide little discriminative information that should be used in combination with more informative features (see Section “Feature combination” below).

The **argumentative structure** information does not seem to improve the performance of the classifiers. The argumentative structure has been assigned based on a trained classifier; thus either mistakes made by the classifier might have impacted the performance or there is simply no overall impact using this kind of classifier.

Table 3 shows the results when the classification attributes are separated according to their **location** (title or abstract - TIAB), for the most promising features so far (Unigrams, Bigrams, Concept names and CUIs, and First level taxonomy) and the best ML algorithm (SVM-perf). Making the distinction of the provenance of the features, either from the title or the abstract of the paper, the performance slightly improves in most of the cases, which is not significant in most of the cases. A larger statistically significant improvement is observed when working with concepts rather than terms.

**Table 3:** **Results of the best performance features (Unigrams, Bigrams, Concepts’ names and CUIs, and First level taxonomy) keeping the source of tokens (either title or abstract), using SVM-perf and a binary representation of features**

	**Precision**	**Recall**	**F-measure**
SVM-perf unigram	0.395	0.654	0.492
SVM-perf bigram	0.414	0.675	0.513*
SVM-perf concepts	0.404	0.646	0.497*
SVM-perf CUIs	0.404	0.643	0.496*
SVM-perf first level taxonomy	0.351	0.653	0.456
SVM-perf TIAB unigram	0.398	0.659	0.496*
SVM-perf TIAB bigram	0.408	0.685	0.512*
SVM-perf TIAB Concepts	0.405	0.656	0.501*
SVM-perf TIAB CUIs	0.407	0.655	0.502*
SVM-perf TIAB first level taxonomy	0.376	0.610	0.465

Results significantly better than unigram (p >0.05) are indicated with *.

In addition, it has been found that **binary features** perform better than **frequency-based features**, as can be seen in Table 4, even though the difference is not significant. This was found as well by Dumais et al. [13] in non-domain specific collections. This might be explained because the abstracts are short, and thus there is a larger variance of term frequencies. We find that using binary features seems to perform better compared to using the term frequency in terms of F-measure, even though it boosts precision at the cost of recall.

**Table 4:** **Binary versus term frequency features using SVMLight and SVM-perf on unigrams and bigrams**

		**Binary**			**TF**	
	**Precision**	**Recall**	**F-measure**	**Precision**	**Recall**	**F-measure**
SVMLight Unigram	0.678	0.302	0.418*	0.694	0.269	0.387
SVMLight Bigram	0.711	0.284	0.406	0.708	0.273	0.394
SVMLight TIAB unigram	0.678	0.302	0.418*	0.700	0.263	0.383
SVMLight TIAB bigram	0.730	0.294	0.420*	0.715	0.268	0.389
SVM-perf Unigram	0.395	0.654	0.492	0.390	0.686	0.497
SVM-perf Bigram	0.414	0.675	0.513	0.442	0.594	0.507
SVM-perf TIAB unigram	0.398	0.659	0.496*	0.401	0.609	0.483
SVM-perf TIAB bigram	0.408	0.685	0.512	0.428	0.611	0.503

For each row, significantly better results (p >0.05) are indicated with *.

When using the **predictions by MTI** as classification features, the performance is below the original MTI performance. MTI system performance is above the other approaches, which is understandable since it has been tuned for MeSH indexing and has specific rules for indexing. Average MTI results are shown in Table 5. In addition to the MTI results, performance of its components are shown. The results for MMI and PRC independently are much lower compared to the MTI results. Performance of individual components of MTI is improved when their predictions are used as features within machine learning algorithms. One difference is the use of machine learning applied to the Check Tags [56], the most commonly used MeSH headings. Another difference is that MTI implements a set of rules produced by interaction with MeSH indexers.

**Table 5:** **MTI results and individual performance of its components MMI (MetaMap + Restrict-to-MeSH) and PRC (PubMed Related citations)**

	**Precision**	**Recall**	**F-measure**
MTI system	0.612	0.499	0.549
MMI	0.556	0.212	0.307
PRC	0.602	0.356	0.447
MMI+PRC	0.600	0.393	0.475

### Feature combination

Results show that performance is dependent on the features and the machine learning algorithms. Overall, unigrams and bigrams seem to be competitive compared to other features with over 0.51 in the F-measure when used with SVM-perf. We could try improving the performance of unigrams by combining them with other feature sets (MTI performance has been shown to improve by combining several sources of information).

A large number of feature combinations could be considered. Based on the results in Table 2, we have selected a limited set of feature combinations using unigrams and bigrams to evaluate the contribution of adding several features. In addition, we show the performance of the learning algorithms when combining all the available features. The results on the combination of features are available from Table 6.

**Table 6:** **Feature combination results**

		**SVM-perf**			**Ada over**	
**Feature combination**	**Prec**	**Rec**	**F1**	**Prec**	**Rec**	**F1**
Unigram	0.395	0.654	0.492	0.528	0.425	0.471
Unigram+CUI	0.409	0.657	0.504*	0.529	0.437	0.479*
Unigram+Meta	0.387	0.672	0.491	0.550	0.405	0.466
Unigram+NP	0.382	0.701	0.495*	0.535	0.424	0.473
Unigram+Taxo	0.403	0.660	0.500*	0.531	0.432	0.477
Unigram+mti	0.448	0.679	0.540*	0.586	0.477	0.526*
Unigram+mmi+prc	0.445	0.677	0.537*	0.583	0.474	0.523*
Unigram+all	0.452	0.689	0.546*	0.600	0.476	0.531*
**Feature combination**	**Prec**	**Rec**	**F1**	**Prec**	**Rec**	**F1**
TIAB+bigram	0.408	0.685	0.512	0.556	0.421	0.479
TIAB+bigram+CUI	0.439	0.688	0.536	0.556	0.435	0.488*
TIAB+bigram+Meta	0.408	0.689	0.513	0.581	0.406	0.478
TIAB+bigram+NP	0.417	0.686	0.518*	0.560	0.422	0.481
TIAB+bigram+Taxo	0.418	0.679	0.518*	0.554	0.412	0.472
TIAB+bigram+mti	0.451	0.701	0.549*	0.604	0.475	0.532*
TIAB+bigram+mmi+prc	0.448	0.699	0.546*	0.607	0.466	0.528*
TIAB+bigram+all	0.470	0.682	0.557*	0.629	0.380	0.474

Results are reported in Precision (Prec), Recall (Rec) and F-measure (F1). Unigrams and bigrams with feature source (either title or abstract, TIAB+bigram) are combined with concepts identifiers (+CUI), meta-data (+Meta), noun phrases (+NP), hypernyms (+Taxo), MTI predictions (+mti), MTI components (mti+prc) and all the features (+all). For each column, results significantly better (p >0.05) than the ones obtained with unigram or TIAB+bigram are indicated with *.

The combination of features has different performance depending on the learning algorithm. AdaBoostM1 with oversampling seems to perform better overall with feature combination than with the bigram baseline. On the other hand, when all the features are combined the performance is lower compared to the baseline. One reason for this is over-fitting of the data due to the large number of features; SVM-perf seems to be more resilient to this problem. This shows as well that the conclusions on the feature experiments depend not only on the feature sets but on the learning algorithms being used. Overall, from the two sets of experiments, better results are obtained in the bigram set, which was already the case in the results without feature combination.

Using noun phases (+NP) shows some non-significant improvement over the unigram and bigram baselines. When using concepts annotated using MetaMap (+CUI) there is a large significant improvement considering the baseline. The improvement is not as important when using concepts from UMLS first level hypernyms (+Taxo) and performance even decreases when using AdaBoostM1. This might be surprising when considering the results only using CUIs, but it may mean as well that CUIs normalize the terms in the citation while adding more general concepts which might make the model less specific to the categorization problem. The article meta-data (+Meta), containing the journal ID, the affiliation and authors, show either a decreased performance or a non-significant improvement.

Considering combining MTI suggestions with either unigrams or bigrams (+MTI), the results are below MTI results, even though SVM-perf with bigrams is closer to MTI’s performance. On the other hand, combining MTI components (+MMI+PRC), we find that the result is better than when considering each component separately. SVM-perf with bigrams+MMI+PRC has a result that is close to MTI’s performance, which is interesting since no manually implemented rules are used compared to MTI’s implementation.

Finally, combining all features improves performance of any individual feature set, except for AdaBoostM1 and bigrams, as mentioned above. Furthermore, when using SVM-perf and combining bigrams with all other features, the results are significantly better than MTI results, showing that it is possible to improve MTI’s performance using automatic methods.

## Discussion

The experiments show that unigrams and bigrams are a strong baseline compared to other more sophisticated features, which is in agreement with previous work. A conceptual representation based on MetaMap shows similar performance compared to unigrams. In addition, combining the features improves the classification performance. When combining all feature sets, even better results than the MTI system are achieved.

The machine learning algorithm has an influence as well on the performance. Looking at the overall results, SVM-perf has the best average performance for the different feature sets (even better the AdaBoostM1 with oversampling). AdaBoostM1 with oversampling performs better than AdaBoostM1 using the natural class distribution. This means that learning algorithms prepared to deal with data set imbalance perform better. We find as well that when all the features are used, AdaBoostM1 with oversampling has decreased performance, which might be due to over-fitting of the learned model. Previous work on feature selection for MeSH categorization has shown a decrease in performance when feature selection is used [29], and it has been seen as well for algorithms like SVM in more general domains [57]. A possible solution might be to increase the size of the training set when a large number of features is being used.

**Figure 1:**
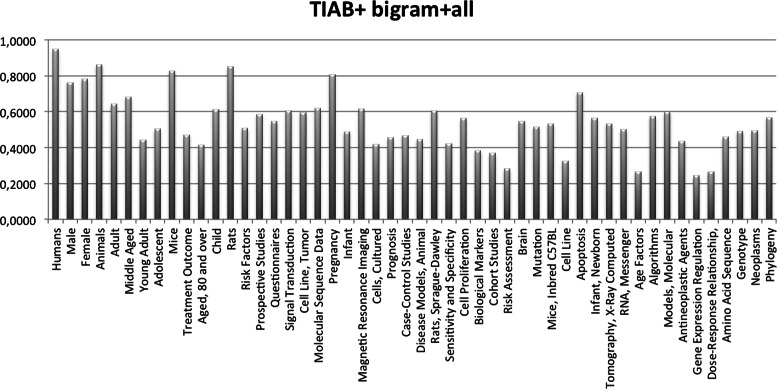
Classification performance per MeSH heading. The figure shows the F-measure for each MeSH heading, when the best combination of features is used for classification (TIAB+bigram+all) and using the best performance ML algorithm (SVM-perf).

Figure 1 shows the F-measure per MeSH heading of the best combination of features (TIAB+bigram+all) and the best performance algorithm (SVM-perf). Our results corroborate previous work that has shown that the performance decreases for the less frequent MeSH headings compared to more frequent ones [11]. We can see this with the most frequent MeSH headings: *Humans*, *Male* and *Female*. Except for a couple of MeSH headings (*Animals* and *Mice*), the performance of the algorithms with respect to these MeSH headings decreases. However, we have observed that using learning algorithms resilient to class imbalance shows improved performance that has a positive impact on less frequent ones.

## Conclusions

Research in MeSH indexing has mostly used the *bag-of-words* representation, leaving other more sophisticated features aside. In this paper, we have studied the feasibility of exploiting the syntactic, semantic and structural properties of texts in order to improve classification of MEDLINE citations with different MeSH headings. Our main conclusion is that, even though traditional features such as unigrams and bigrams have strong performance compared to other features, it is possible to combine them to effectively improve the performance of the *bag-of-words* representation for MeSH indexing. The combination allows improving the performance over the MTI system, that has been shown to be a hard baseline to improve on [28]. We have also found that the selection of the learning algorithm has an influence in the overall performance of the system. Algorithms that are more resilient to the imbalance of the data set show improved performance.

As future work, we plan to take further features into consideration, including the part-of-speech, using automatic summaries from PMC full text articles instead of the abstracts for extracting the classification features, exploiting more in depth the argumentative structure of the abstract, for instance, by including as features only the tokens from relevant sections, and exploring additional features from MEDLINE or full text articles. We have not investigated the combination of learning algorithms in this work, which has previously shown to improve performance [56]. We did not investigate the selection of MeSH headings according to the best performing combination of features and methods [11]. Recent work using deep learning for MeSH indexing [58] shows promising results and could be considered to obtain a better set of features automatically. All this could be explored as future work. Furthermore, since over-fitting is a problem with some learning algorithms and feature selection seems to decrease performance [29], larger citation sets could be considered in further experiments.

## Additional file

Additional file 1
**Extended results.** These extended results are in an Excel file. The first four sheets (*SVMLight*, *SVM_perf*, *AdaBoostM1* and *AdaBoostM1 Oversampling*) contain detailed results for the feature sets for each of the algorithms for the different MeSH headings. Combination of feature sets are available from the sheets *Feature Combination SVM_perf* and *Feature Combination Ada Over*. Results are measured in Precision (P), Recall (R) and F-measure (F1). The sheet *Overall results* contains a summary of the average performance of the experiments.

## References

[1] Sebastiani F (2002). Machine learning in automated text categorization. ACM Comput Surveys (CSUR).

[2] Jimeno-Yepes A, Wilkowski B, Mork JG, Lenten EV, Fushman DD, Aronson AR. A bottom-up approach to MEDLINE indexing recommendations. In: Proceedings of the AMIA Annual Symposium: 2011. p. 1583–92. PMC3243198 22195224

[3] Scott S, Matwin S. Feature engineering for text classification. In: ICML, Volume 99. Citeseer: 1999. p. 379–88.

[4] Bodenreider O (2004). The unified medical language system (UMLS): integrating biomedical terminology. Nucleic Acids Res.

[5] Bodenreider O, Nelson SJ, Hole WT, Chang HF. Beyond synonymy: exploiting the UMLS semantics in mapping vocabularies. In: Proceedings of the AMIA symposium. American Medical Informatics Association: 1998. p. 815. PMC2232139 9929332

[6] Ruch P, Tbahriti I, Gobeill J, Aronson AR. Argumentative feedback: a linguistically-motivated term expansion for information retrieval. In: Proceedings of the COLING/ACL on Main conference poster sessions. Association for Computational Linguistics: 2006. p. 675–82.

[7] Ruch P, Boyer C, Chichester C, Tbahriti I, Geissbühler A, Fabry P (2007). Using argumentation to extract key sentences from biomedical abstracts. Int J Med Informatics.

[8] Jimeno-Yepes A, Mork JG, Aronson AR. Using the argumentative structure of scientific literature to improve information access. In: Proceedings of the 2013 Workshop on Biomedical Natural Language Processing (BioNLP 2013): 2013. p. 102–10.

[9] Jimeno-Yepes AJ, Sticco JC, Mork JG, Aronson AR. GeneRIF indexing: sentence selection based on machine learning. BMC Bioinf. 2013; 14:171. 10.1186/1471-2105-14-171 PMC3687823 23725347

[10] Rennie JD, Shih L, Teevan J, Karger DR. Tackling the poor assumptions of naive bayes text classifiers. In: ICML, Volume 3. Washington DC: 2003. p. 616–23.

[11] Jimeno Yepes A, Mork JG, Aronson AR. Comparison and combination of several MeSH indexing approaches. In: AMIA annual symposium proceedings. Volume 2013. American Medical Informatics Association: 2013. PMC3900212 24551371

[12] Apte C, Damerau F, Weiss SM, Apte C, Damerau F, Weiss SM (1994). Automated learning of decision rules for text categorization. ACM Trans Inf Syst.

[13] Dumais S, Platt J, Sahami M, Heckerman D. Inductive learning algorithms and representations for text categorization. In: ACM Transactions on Information Systems. ACM Press: 1998. p. 148–55.

[14] Fuhr N, Hartmann S, Lustig G, Schwantner M, Tzeras K, Knorz G. AIR/X - a rule-based multistage indexing system for large subject fields. In: Proceedings of RIAO’91: 1991. p. 606–23.

[15] Schutze H, Hull DA, Pedersen JO. A comparison of classifiers and document representations for the routing problem. In: Annual ACM Conference on Research and Development in Information Retrieval - ACM SIGIR. ACM: 1995. p. 229–37.

[16] Bekkerman R, Allan J. Using Bigrams in Text Categorization; 2003.

[17] Lewis DD (1992). An evaluation of phrasal and clustered representations on a text categorization task. Proceedings of the 15th Annual International ACM SIGIR Conference on Research and Development in Information Retrieval, SIGIR ’92.

[18] Pang B, Lee L, Vaithyanathan S. Thumbs up? Sentiment classification using machine learning techniques. In: Proceedings of EMNLP: 2002. p. 79–86.

[19] Furnkranz J, Mitchell T, Riloff E. A case study in using linguistic phrases for text categorization on the www. In: Working Notes of the AAAI/ICML Workshop on Learning for Text Categorization. AAAI Press: 1998. p. 5–12.

[20] Gabrilovich E, Markovitch S. Feature generation for text categorization using world knowledge. In: IJCAI 05: 2005. p. 1048–53.

[21] Aronson AR, Mork JG, Gay CW, Humphrey SM, Rogers WJ (2004). The NLM indexing initiative’s medical text indexer. Medinfo.

[22] Sohn S, Kim W, Comeau DC, Wilbur WJ (2008). Optimal training sets for bayesian prediction of MeSH®; assignment. J Am Med Informatics Assoc.

[23] Yetisgen-Yildiz M, Pratt W. The effect of feature representation on MEDLINE document classification. In: AMIA annual symposium proceedings. Volume 2005. American Medical Informatics Association: 2005. p. 849. PMC1560754 16779160

[24] Hersh W, Buckley C, Leone T, Hickam D. OHSUMED: An interactive retrieval evaluation and new large test collection for research. In: SIGIR 94. Springer: 1994. p. 192–201.

[25] Kouramajian V, Devadhar V, Fowler J, Maram S. Categorization by reference: a novel approach to MeSH term assignment. In: Proc Annu Symp Comput Appl Med Care: 1995. p. 878–82. PMC2579219 8563418

[26] Ortuño FM, Rojas I, Andrade-Navarro MA, Fontaine JF. Using cited references to improve the retrieval of related biomedical documents. BMC Bioinf. 2013; 14:113. 10.1186/1471-2105-14-113 PMC3618341 23537461

[27] Herskovica JR, Cohena T, Subramanian D, Iyengara MS, Smitha JW, Bernstama EV (2011). MEDRank: Using graph-based concept ranking to index biomedical texts. Int J Med Informatics.

[28] BioASQ workshop (accessed May 1st, 2014). [http://www.bioasq.org/workshop1/schedule]

[29] Spolaor N, Tsoumakas G. Evaluating feature selection methods for multi-label text classication. In: BioASQ workhsop: 2013.

[30] Mork JG, Jimeno Yepes A, Aronson AR. The NLM medical text indexer system for indexing biomedical literature. In: BioASQ workhsop: 2013.

[31] Cohen WW (1995). Learning to classify English text with ILP methods. Advances in inductive logic programming.

[32] Névéol A, Shooshan S, Claveau V. Automatic inference of indexing rules for MEDLINE. BMC Bioinf. 2008; 9(Suppl 11):S11. 10.1186/1471-2105-9-S11-S11 PMC2586750 19025687

[33] Aronson A. Effective mapping of biomedical text to the UMLS Metathesaurus: the MetaMap program. In: Proceedings of the AMIA Symposium: 2001. p. 17–21. PMC2243666 11825149

[34] Aronson AR, Lang FM (2010). An overview of MetaMap: historical perspective and recent advances. J Am Med Informatics Assoc.

[35] MetaMap (accessed March 13th, 2014). [http://metamap.nlm.nih.gov/]

[36] Lin J, Wilbur W.PubMed related articles: a probabilistic topic-based model for content similarity. BMC Bioinf. 2007; 8:423. 10.1186/1471-2105-8-423 PMC2212667 17971238

[37] Fung K, Bodenreider O. Utilizing the UMLS for semantic mapping between terminologies. In: Proceedings of the AMIA Annual Symposium: 2005. PMC1560893 16779043

[38] Tan CM, Wang YF, Lee CD (2002). The use of bigrams to enhance text categorization. Inf Process Manage.

[39] UMLS (Unified Medical Language System) (accessed March 13th, 2014). [http://www.nlm.nih.gov/research/umls/]

[40] UMLS Reference Manual (accessed March 13th, 2014). [http://www.ncbi.nlm.nih.gov/books/NBK9676/]

[41] Smith L, Rindflesch T, Wilbur WJ (2004). MedPost: a part-of-speech tagger for bioMedical text. Bioinformatics (Oxford, England).

[42] Jimeno Yepes A, Mork JG, Aronson AR. Identifying publication types using machine learning. In: BioASQ workhsop: 2013.

[43] 2014 Vocabulary Density Study Datasets (accessed Dec 16th, 2014). [http://ii.nlm.nih.gov/DataSets/index.shtml#2014_VocabDensity]

[44] Wang X, Chen R, Jia Y, Zhou B. Short Text Classification using Wikipedia Concept based Document Representation. In: Proceedings of the 2013 International Conference on Information Technology and Applications: 2013. p. 471–4.

[45] Ruch P, Cohen G, Ehrler F, Müller H, Coray G, Ghorbel H, et al.Report on the TREC 2003 Experiment: genomic track. In: TREC: 2003. p. 756–61.

[46] Tbahriti I, Chichester C, Lisacek F, Ruch P.Using argumentation to retrieve articles with similar citations: An inquiry into improving related articles search in the MEDLINE digital library. Int J Med Informatics. 2005:75. 10.1016/j.ijmedinf.2005.06.007 16165395

[47] Plaza L, Carrillo-de Albornoz J. Evaluating the use of different positional strategies for sentence selection in biomedical literature summarization. BMC Bioinf. 2013; 14:71. 10.1186/1471-2105-14-71 PMC3648362 23445074

[48] Ripple AM, Mork JG, Knecht LS, Humphreys BL. A retrospective cohort study of structured abstracts in MEDLINE, 1992-2006. J Med Library Assoc. 2011; 99(2):160. 10.3163/1536-5050.99.2.009 PMC3066587 21464855

[49] Plaza L, Díaz A, Gervás P. (2011). A semantic graph-based approach to biomedical summarisation. Artif Intelligence Med.

[50] MTI ML 2013 data set (accessed May 1st, 2014). [http://ii.nlm.nih.gov/DataSets/index.shtml#2013_MTI_ML]

[51] Joachims T. Text categorization with support vector machines: learning with many relevant features: Springer; 1998.

[52] MTI ML site (accessed May 1st, 2014). [http://ii.nlm.nih.gov/MTI_ML]

[53] Joachims T. A support vector method for multivariate performance measures. In: Proceedings of the 22nd international conference on Machine learning. ACM: 2005. p. 377–84.

[54] SVM-perf site (accessed May 1st, 2014). [http://www.cs.cornell.edu/People/tj/svm_light/svm_perf.html]

[55] Cohen PR. Empirical methods for artificial intelligence. Volume 139: MIT press Cambridge; 1995.

[56] Jimeno-Yepes A, Mork JG, Demner-Fushman D, Aronson AR (2012). A one-size-fits-all indexing method does not exist: automatic selection based on meta-learning. JCSE.

[57] Joachims T. Svmlight: Support vector machine. SVM-Light Support Vector Machine, University of Dortmund. 1999; 19(4). http://svmlight.joachims.org/.

[58] Jimeno Yepes A, MacKinlay A, Bedo J, Garnavi R, Chen Q. Deep belief networks and biomedical text categorisation. In: Australasian Language Technology Association Workshop: 2014. p. 123.

